# Exploration of the minimum necessary FVIII level at different physical activity levels in pediatric patients with hemophilia A

**DOI:** 10.3389/fped.2022.1045070

**Published:** 2022-11-01

**Authors:** Di Ai, Kun Huang, Gang Li, Yingzi Zhen, Xinyi Wu, Ningning Zhang, Aihua Huo, Zhenping Chen, Runhui Wu

**Affiliations:** ^1^ Hematology Center, Beijing Children's Hospital, Capital Medical University, National Center for Children's Health, Beijing, China; ^2^ Hematologic Disease Laboratory, Beijing Pediatric Research Institute, Beijing Children's Hospital, Capital Medical University, Beijing, China; ^3^ Department of Radiology, Beijing Children's Hospital, Capital Medical University, National Center for Children's Health, Beijing, China

**Keywords:** hemophilia A, children, physical activity, FVIII level, hemarthrosis

## Abstract

**Background:**

Physical activity can increase joint stability and reduce the risk of injury in hemophilia patients. There is limited clinical data on target trough FVIII levels during physical activity in hemophilia A patients. Hence, this study aimed to explore the target trough FVIII level required to avoid bleeding during different physical activities in hemophilia A patients.

**Methods:**

Patients with severe or moderate hemophilia A, who underwent pharmacokinetics (PK) tests at our center were enrolled in this study. Physical activities and clinical information such as bleeding were recorded. The FVIII level during physical activity was calculated by the WAPPS-Hemo.

**Results:**

A total of 105 patients were enrolled in this study. A total of 373 physical activities were recorded, of which 57.6% (215/373) was low-risk activities and the remaining 42.4% (158/373) was medium-risk activities. Most common physical activities were bicycling (59.0%), swimming (43.8%), running (48.6%), and jumping rope (41.0%). The FVIII trough level of low-risk physical activity was 3.8 IU/dl (AUC = 0.781, *p *= 0.002) and moderate-risk physical activity was 7.7 IU/dl (AUC = 0.809, *p *< 0.001). FVIII trough levels [low-risk activities: 6.1 (3.1, 13.2) IU/dl vs. 7.7 (2.3, 10.5) IU/dl, moderate-risk activities: 9.6 (5.8, 16.9) IU/dl vs. 10.2 (5.5, 11.0) IU/dl] were not statistically different between the mild arthropathy group and the moderate-severe arthropathy group. Multiple bleeding risk tended to increase with physical activities classified as moderate-risk (OR [95% CI]: 3.815 [1.766–8.238], *p *= 0.001).

**Conclusion:**

The minimum necessary FVIII level increased with higher risk physical activity, irrespective of arthropathy.

## Essentials

1. Lack of clinical data on target trough FVIII levels during physical activity.
2. The FVIII level during physical activity was calculated by the WAPPS-Hemo.
3. Higher-risk physical activity correlated with greater probability of bleeding and FVIII levels are protective factors for not bleeding during exercise.
4. The target trough FVIII increased with risk physical activity increases no matter if arthropathy.

## Introduction

Hemophilia A is a rare inherited bleeding disorder caused by the deficiency or dysfunction of coagulation factor VIII (FVIII). As the standard treatment for hemophilia, prophylaxis can reduce bleeding events, prevent joint damage, and maintain normal joint structure and function (1). Previous studies have demonstrated the key issues in prophylaxis of hemophilia A, such as the variability of individualized pharmacokinetics, bleeding phenotype and joint vulnerability (2). Thus, individualized prophylaxis needs to consider the individual's bleeding pattern, condition of the musculoskeletal system, level of physical activity and the pharmacokinetic profiles (3).

With the widespread prophylaxis, physical activity levels of patients with hemophilia have increased (4). With appropriate FVIII level, the benefits of physical activity outweigh the risk of bleeding (5). Lack of physical activity will lead to increased body fat and decreased leg muscle mass (6). Regular physical activity can increase joint stability and reduce the risk of injury (7). According to the World Federation of Hemophilia (WFH) recommendations, physical activity can improve patients' quality of life and clinical outcomes (1). Although there is expert consensus on the recommended FVIII levels during sports (8), clinical data on target trough FVIII levels during physical activity is very limited.

Recently, we conducted individualized prophylaxis in pediatric patients with severe hemophilia A, and achieved good clinical outcomes (9). We collected the details of physical activity and calculated the FVIII level in different physical activities, to explore the FVIII level required to avoid bleeding during different physical activities.

## Materials and methods

### Ethics

The study was approved by the Ethics Committee of Beijing Children's Hospital. All patients and/or legally authorized guardians gave written informed consent.

### Study design

This was a retrospective, cohort, observational study. The patients with severe or moderate hemophilia A, who underwent pharmacokinetics (PK) tests at our center, were enrolled at Beijing Children's Hospital between June 2018 and June 2021.

### Patients

Inclusion criteria: Patients aged ≤18 years with severe or moderate hemophilia A (FVIII activity <5 IU/dl); Patients taking plasma-derived or recombinant FVIII concentrates for prophylaxis and the same treatment regimen for >3 months; Patients who underwent PK tests at our center. Exclusion criteria: Patients with an inhibitor titer ≥0.6 Bethesda Unit (BU)/ml (confirmed by two separate tests); with other concomitant bleeding or chronic disorders.

### PK evaluation and FVIII level calculation

PK analysis was performed by Web-Accessible Population Pharmacokinetic System (WAPPS-Hemo). A five-point assay was utilized (Pre-dose, 1 h, 9 h, 24 h, 48 h) (10). FVIII levels were determined by one-stage assay. According to patients' routine dose and frequency, the FVIII level during physical activity was calculated by the WAPPS-Hemo.

### Clinical data collection

Baseline FVIII:C levels, age, von Willebrand factor antigen and blood group were obtained from the medical records. Every time the patients came for follow-up, we recorded the prophylaxis regime, type of physical activity, time of physical activity, bleeding, and the time of infusion of the FVIII, from the parents. We recorded all physical activities of the patients. Their detailed information was obtained from their paper-based or online record, which would be confirmed at their monthly visit at our center. If the patient performed the same physical activity at different times, we chose the physical activity with the lowest FVIII level when the patient had no bleeding.

### Definitions

Risk associated with physical activities: In this study, the patient's physical activity was classified into low-risk (safe, safe-to-moderate), moderate-risk (moderate, moderate-to-dangerous), and high-risk (dangerous) groups based on the National Hemophilia Foundation (NHF) definition (11).

Patients were considered overweight, or obese according to the guidelines for the evaluation, treatment and prevention of childhood obesity in China (12). Overweight was defined as BMI ≥ 2SD of the median value of age and gender, and obesity was defined as BMI ≥ 3SD of the corresponding population.

The six index joints were evaluated by ultrasound, as previously described by Zhang et al. (13). According to the ultrasound results, the patients were divided into the mild arthropathy group and the moderate-severe arthropathy group. Effusion/bleeding and synovial change were mild arthropathy. Hemosiderin deposition and bone/cartilage change were moderate-severe arthropathy.

### Statistical analysis

The statistical analyses were performed using GraphPad Prism for Mac (Version 9.0.1). Data were reported as median (upper quartile, lower quartile) with range. For comparison of the differences between the groups, the Mann–Whitney *U*-test was utilized. Logistic regression models were used to evaluate the association between physical activity levels, FVIII level, and activity-related bleeding risk of patients. Cutoff value was obtained by receiver operating characteristic (ROC) curve. A *p-*value <0.05 indicated a statistically significant difference.

## Results

### Patients

A total of 105 patients were enrolled. Their median age was 8.5 (5.7, 10.1) years. The prophylaxis trough FVIII level of most patients (61.9%) was between 1 and 5 IU/dl. Moreover, 35.2% patients were overweight or obese. The characteristics of the 105 patients are shown in Table 1.

**Table 1:** Characteristics of the patients included in this study.

	Median (quartiles)	Mean (range)	Number (%)
**Hemophilia severity**			
Severe			94 (89.5%)
Moderate			11 (10.5%)
Age (year)	8.5 (5.7, 10.1)	8.3 (2.6–15.3)	
Body weight (kg)	30.0 (22.0, 43.0)	33.1 (12.0–85.0)	
Body mass index (kg/m2)	16.6 (15.3, 20.4)	17.6 (12.8–29.6)	
Overweight			25 (23.8%)
Obesity			12 (11.4%)
von Willebrand factor antigen (%)	95.9 (75.5, 120.8)	98.9 (42.0–242.0)	
Dose, IU/kg	22.7 (17.3, 31.8)	25.4 (10.0–50.0)	
Dosing frequency, number of times/weeks	3.5 (2.3, 3.5)	3.0 (1–3.5)	
Trough level			
<1 IU/dl			24 (22.9%)
1–3 IU/dl			39 (37.1%)
3–5 IU/dl			26 (24.8%)
>5 IU/dl			16 (15.2%)

### Physical activities and FVIII activity levels

In total, 373 physical activities were recorded. Meanwhile, 28 (26.7%) patients skipped physical education lessons in school. Among these physical activities, 57.6% (215/373) was low-risk activities, while the other 42.4% (158/373) was medium-risk activities. No patient engaged in high-risk activities. A total of 19 patients had 34 joint bleeding after physical activity, of which 23 (67.6%) were ankle bleeding, nine (26.5%) were knee bleeding, and two (5.9%) were elbow bleeding.

**Figure 1:**
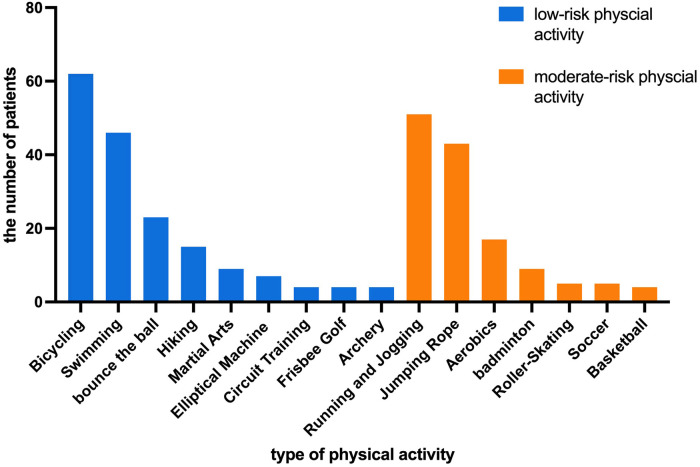
The types of physical activities performed by the patients.

The most common physical activities were bicycling, swimming, running, and jumping rope. Among the 105 patients, 62 (59.0%) patients performed bicycling, 51 (48.6%) patients performed running, 46 (43.8%) patients performed swimming and 43 (41.0%) patients performed jumping rope. The types of activities are shown in Figure 1. The median weekly frequency of physical activity participation was 2 (1, 3) times.

There were 11(5.1%) joint bleeding after low-risk physical activity and 23 (14.6%) joint bleeding after moderate -risk physical activity. Analysis of FVIII trough levels during physical activity without bleeding showed that the median FVIII trough levels of low-risk activities were 6.1 IU/dl (interquartile: 2.9, 10.9) and moderate-risk activities were 9.1 IU/dl (interquartile: 5.5, 16.5). There was a statistical difference between the FVIII levels of low-risk and moderate-risk activities (*Z* = −4.213, *p *< 0.001). FVIII trough levels of low-risk activities were 2.7 IU/dl (interquartile: 1.1, 3.7) and moderate-risk activities were 3.4 IU/dl (interquartile: 1.1, 7.5) in physical activity with bleeds.

**Figure 2:**
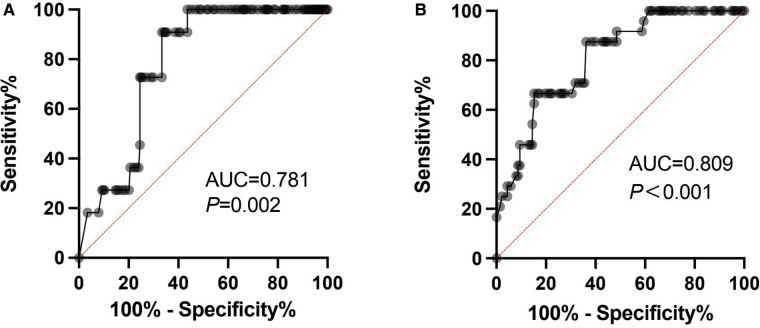
The FVIII trough level ROC curves.

The FVIII trough level ROC curve was obtained based on whether bleeding occurred after physical activity. The cutoff of FVIII trough level was 3.8 IU/dl (AUC = 0.781, *p *= 0.002, Sensitivity = 90.9%, Specificity = 66.7%) in patients with low-risk physical activity and 7.7 IU/dl (AUC = 0.809, *p *< 0.001, Sensitivity = 87.5%, Specificity = 63.8%) in patients with moderate-risk physical activity (Figure 2).

### Arthropathy and FVIII trough levels (TTL) at physical activity

Among the 105 patients, five (4.8%) patients had no abnormality on ultrasound in six target joints, 77 (73.3%) patients had mild arthropathy in at least one joint, and 23 (21.9%) patients had moderate-severe arthropathy in at least one joint. The patients' characteristics of the two groups are shown in Table 2. The age of the mild arthropathy group was younger than the moderate-severe arthropathy group (*p *< 0.001). When performing low-risk physical activities, the medium FVIII trough level of the mild arthropathy group was 6.1 IU/dl and the moderate-severe arthropathy group was 7.7 IU/dl. When performing low-risk physical activity, the medium FVIII trough level of the mild arthropathy group was 9.6 IU/dl and the moderate-severe arthropathy group was 10.2 IU/dl. The FVIII trough levels were not statistically different between the two groups.

**Table 2:** Characteristics of the mild arthropathy and moderate-severe arthropathy groups.

	Mild arthropathy	Moderate -severe arthropathy	*p*-value
Age, years	7.6 (5.3, 9.6)	9.8 (9.2, 13.0)	<0.001
BMI	16.1 (15.1, 19.2)	18.8 (15.7, 21.1)	0.027
vWF	97.0 (77.7, 117.7)	75.9 (70.7, 118.3)	0.271
Trough FVIII levels for prophylaxis	2.1 (1.0, 4.0)	2.3 (0.5,4.7)	0.912
Low-risk activities FVIII levels	6.1 (3.1, 13.2)	7.7 (2.3, 10.5)	0.917
Moderate-risk activities FVIII levels	9.6 (5.8, 16.9)	10.2 (5.5, 11.0)	0.512

### Risk of physical activity-related bleeding

As the univariate analysis showed, only the FVIII level during activity (*p* < 0.001) and risk of activity (*p* = 0.002) demonstrated significant influence on bleeds. While the influence other factors like age (*p* = 0.884), the numbers of arthropathy (*p* = 0.145), vWF:Ag level (*p* = 0.275), FVIII trough level of prophylaxis (*p* = 0.085) and the degree of arthropathy (*p* = 0.314) showed no statistical significance. Logistic regression analysis showed that FVIII levels during activity and activity risk were independent risk factors of bleeding after physical activity. Multiple bleeding risk tended to increase with physical activities classified as moderate-risk (OR [95% CI]: 3.815 [1.766–8.238], *p *= 0.001) (Table 3).

**Table 3:** Logistic regression models were used to evaluate the association between physical activity levels, FVIII level, and activity-related bleeding risk of patients.

	Univariate analysis		Multiple logistic regression analysis		
	Test statistic	*p*-value	OR	95% CI	*p*-value
Age	−0.146	0.884			
FVIII level during activity	−4.155	<0.001	0.924	0.865–0.986	0.017
Activity risk low-risk vs. moderate- risk	9.798	0.002	3.815	1.766–8.238	0.001
the numbers of arthropathy	−1.458	0.145			
Degree of arthropathy	1.015	0.314			
vWF: Ag	−1.092	0.275			
BMI	−1.587	0.113			
Normal vs. overweight	3.375	0.066			
FVIII trough level of prophylaxis	−1.723	0.085			

## Discussion

In the past few decades, the prophylaxis of hemophilia A has continually evolved, due to which children with hemophilia can have a normal life, including increased participation in sports. Physical activity is necessary for children with hemophilia, as it can provide better joint protection through improved muscle mass and strength, wider joint range of motion and enhanced proprioception (14). For children above six years of age, expert consensus recommends minimum 60 min of exercise per day (15). However, the FVIII level should be maintained to avoid bleeding. Although the expert consensus has given recommendations, related clinical data remains very limited. Thus, this study was designed to elucidate the target trough FVIII level while performing different physical activities.

Physical activity has been recommended for patients with hemophilia since the mid-1970s. According to Buxbaum et al., in the current social environment and with medical support, patients with hemophilia can be as physically active as their normal peers (16). A recent systematic review showed that physical activity levels varied greatly among heterogeneous samples of patients with hemophilia (4). In this study, patients participated in at least one physical activity besides routine walking. The most common activities were bicycling, swimming, running, and rope jumping. Heijnen's research (17) showed that soccer, swimming, cycling and gymnastics were most common in Dutch population. The proportion of soccer in Heijnen's study was 21%, but the number was only 4.8% in our patients. Given that China is a developing country with limited sources, it is important to estimate the low trough FVIII level in routine prophylaxis. Since many elementary schools' require tests of jumping rope, it accounts for a high proportion in our study. In our study, 26.7% patients chose not to participate in physical education. A British study of boys aged 6–17 years with hemophilia A or B (*n* = 84) reported 90.5% participated in regular sports activity (18). Most patients participated in low-risk activities, and no patient participated in high-risk activities.

Since all the enrolled patients were children, the over-cautiousness of their parents made it less likely for them to encourage their child's participation in athletics. Previous studies have revealed that sports could protect joints (19). Regular physical activity is also conducive to maintaining normal weight. Overweight and obesity are higher among patients in our study as compared to the boys aged 7–18 years in urban China (20). Normal weight is good for health, and can also reduce the dose of FVIII and joint bleed. Gupta et al. evaluated the association of BMI with joint bleeds, and patients who were overweight and obese had higher rate of joint bleeds than patients who were normal/underweight [aIRR 1.05 (95% CI: 0.98; 1.13), 1.11 (95% CI: 1.04; 1.20)] (21). Hence, patient education should be strengthened and children with hemophilia should be encouraged to participate in appropriate physical activity.

Previous studies have examined the relationship between physical activity and bleeding outcomes in children with hemophilia. Tiktinsky et al. evaluated the association between risk of injury and the level of activity, and revealed that strenuous activity was associated with an increased risk of bleeding (22). Broderick et al. reported that compared with inactivity and category 1 activity (e.g., swimming), category 2 activities (e.g., basketball) were associated with a transient increase in the risk of bleeding [OR 2.7 (95% CI: 1.7; 4.8)]. Category 3 activities (e.g., wrestling) were associated with a greater transient increase in risk [OR 3.7 (95% CI: 2.3; 7.3)] (5). Barbara et al. showed that activities with a high risk of collision lead to an increased risk of bleeding (23).

Similar to previous studies, the minimum necessary FVIII level increased with higher risk physical activity irrespective of arthropathy in our study. Many studies have shown that higher plasma factor levels are associated with lower risk for bleeding. Higher activity-related bleeding risk has been observed in children with lower FVIII levels (trough FVIII level ≤5%). Each increment of 1% in the FVIII level with treatment before sports correlates with a decreased bleeding risk by 2% (5). Compared to patients with severe hemophilia, non-severe type patients had higher participation in high-risk sports (65% vs. 48%, *p *= 0.05) (24). In this study, we explored the minimum necessary FVIII level during different physical activities. The ROC analysis showed that the cutoff value of minimum FVIII level in low-risk physical activities was 3.8%, which was consistent with Antony et al. who showed expert elicitation exercise (4.1%) (8). However, the minimum FVIII level in moderate-risk physical activities was 7.7% in our study, while Antony et al. found that the minimum level was 11.4% (8). This may be attributed to the low overall participation of children in physical activity in China. A Dutch study in young patients with hemophilia (age: 6–18 years) reported that the median weekly sports frequency was 3 (25). In our study, the median weekly frequency of physical activity participation was 2. In order to obtain more accurate results, a more detailed evaluation is needed.

A higher factor level at the time of injury is a predictive factor of bleeding events (26). A cohort study showed that for every hour spent at <1 IU/dl FVIII levels, the risk of bleeding increased by 1.4–2.2% (27). In this study, we found that higher-risk physical activity was correlated with greater probability of bleeding, and FVIII level was a protective factor against bleeding during exercise. The risk of bleeding may be influenced by the levels of joint morbidity. A recent study showed that higher Patterson score in all joints was associated with higher median joint bleeding (28). However, there was no difference in FVIII levels between different degrees of joint damage while doing physical activity in our study. Arthropathy is not associated with bleeding after physical activity. A recent systematic review (29) found that plasma factor levels, history of bleeds, and physical activity are risk factors for bleeding.

The limitations of this study were that some clinical data (including time of physical activity and the time of infusion of the coagulation factor) came from records of the parents. Children with hemophilia typically refrain from high-risk physical activity. In the future, further research is needed to confirm the findings.

## Conclusion

The minimum necessary FVIII level increased with higher risk physical activity irrespective of arthropathy in this study. These findings may be used to guide patients with hemophilia in physical activities.

## Data Availability

The raw data supporting the conclusions of this article will be made available by the authors, without undue reservation.
